# Developing a differential attainment e-learning course for consultants who supervise trainees within Oxleas NHS Foundation Trust

**DOI:** 10.1192/bjo.2021.445

**Published:** 2021-06-18

**Authors:** Naomi Tomlinson, Bani Kahai, Femi Balogun

**Affiliations:** 1 SLaM; 2 Oxleas NHS Foundation Trust, London

## Abstract

**Aims:**

To create an e-learning course to promote awareness of differential attainment and encourage supervisors to reflect on their own experiences and practice.

**Method:**

Funding was gained from Health Education England to create the e-learning course. A questionnaire was constructed to gauge baseline knowledge and attitudes towards differential attainment amongst the consultant body. All consultants attending a local Faculty Day were asked to respond, and following this an explorative discussion on the topic was chaired by the authors. The results of the survey were collated and free-text answers were coded thematically. In parallel, information from academic publications and professional resources was gathered and summarised. A script was created with support from web developers Kineo and was refined over several drafts. The e-learning module was published on the Oxleas learning environment on the 10th February 2021.

**Result:**

34 supervisors responded to our questionnaire. 75% had heard of DA, with 45% identifying personal experience of it. However only 35% identified it as a problem in their work place and 39% did not consider it in their clinical practice. Thematic analysis of free text comments revealed three main themes – emotions and experiences associated with differential attainment, a desire for increased training and a desire for more open discussions with struggling trainees. Some answers also revealed poor or incomplete understanding of the topic.

From the questionnaire and the literature, four key areas were identified – defining differential attainment, describing the scope of the problem, challenging misconceptions about differential attainment and the role of the social network in attainment. These four areas became section titles for the e-learning course.

**Conclusion:**

There is an appetite for information pertaining to differential attainment amongst our consultant body. A trainer facing e-learning course was created to promote awareness and reflection on current practice. Evaluation of the impact of the module is ongoing. The course is being shared with the confederation of South London local education providers.

